# Isolation of midgut escape mutants of two American genotype dengue 2 viruses from *Aedes aegypti*

**DOI:** 10.1186/1743-422X-10-257

**Published:** 2013-08-12

**Authors:** Cynthia CH Khoo, Jeffrey B Doty, Nicole L Held, Ken E Olson, Alexander WE Franz

**Affiliations:** 1 Arthropod-borne and Infectious Diseases Laboratory, Department of Microbiology, Immunology & Pathology, Colorado State University, Fort Collins, CO 80523, USA; 2 Department of Veterinary Pathobiology, University of Missouri, 303 Connaway Hall, Columbia, MO 65211, USA

**Keywords:** Dengue virus type 2, American genotype, *Aedes aegypti*, Midgut escape mutant, Amino acid substitution

## Abstract

**Background:**

Several studies have shown that American genotype dengue 2 viruses (DENV2) have reduced viral fitness in the mosquito vector, *Aedes aegypti*, compared to other DENV2 genotypes. Diminished replication efficiency or inability to efficiently traverse membrane barriers encompassing organs such as the midgut or salivary glands are considered major factors negatively impacting viral fitness in the mosquito.

**Results:**

We analyzed the vector competence of *Ae. aegypti* for two American DENV2 strains, QR94 and PR159 originating from Mexico and Puerto-Rico, respectively. Both strains infected mosquito midguts following acquisition of infectious bloodmeals. However, DENV2-QR94 and DENV2-PR159 poorly disseminated from the midgut at 7 or 14 days post-bloodmeal (pbm). We detected one virus isolate, EM33, among 31 DENV2-QR94 infected mosquitoes, and one isolate, EM41, among 121 DENV2-PR159 infected mosquitoes, generating high virus titers in mosquito carcasses at 7 days pbm. In oral challenge experiments, EM33 and EM41 showed midgut dissemination rates of 40-50%. Replication efficiency of EM41 in secondary mosquito tissue was similar to that of a dissemination-competent control strain, whereas the replication efficiency of EM33 was significantly lower than that of the control virus. The genome sequence of DENV2-QR94 encoded seven unique amino acids (aa), which were not found in 100 of the most closely related DENV2 strains. EM33 had one additional aa change, E202K, in the E protein. DENV2-PR159 encoded four unique aa residues, one of them E202K, whereas EM41 had two additional aa substitutions, Q77E in the E protein and E93D in NS3.

**Conclusions:**

Our results indicate that the midgut of *Ae. aegypti* acts as a selective sieve for DENV2 in which genetically distinct, dissemination-competent virus variants are rapidly selected from the viral quasispecies to be transmitted to vertebrates.

## Background

Dengue is world-wide the most important arthropod-borne viral disease, infecting an estimated 50 million people annually, of which around 500,000 develop severe hemorrhagic fever/dengue shock syndrome [1]. The estimated mortality amounts to ~20,000 cases per annum [2]. The four serotypes of dengue virus (*Flaviviridae; Flavivirus*; *dengue virus type 1–4* [DENV1-4]) are transmitted primarily by the yellow fever mosquito, *Aedes aegypti*[3]. Urban dengue circulates exclusively between mosquitoes and humans without the need of another (animal) amplification host. Infected humans typically develop high viremia, which enables mosquitoes to readily acquire the virus from their hosts for further transmission.

Following acquisition of an infectious bloodmeal, DENV particles enter the midgut lumen [4]. Viruses that are in close proximity to the epithelial cell lining of the midgut invade the epithelial cells and start replicating in those cells. After four to seven days of replication, the virus starts to disseminate from the midgut epithelial cells to secondary tissue such as nerve tissue, hemocytes, fat body, reproductive tissue, and eventually the salivary glands [4-6]. Once the salivary glands are infected and DENV particles are released into the saliva ducts, the mosquito is able to transmit the virus to a new host. In a number of studies it has been shown that the mosquito midgut is a critical organ determining vector competence for DENV [7,8]. The virus can encounter a midgut infection barrier (MIB), which restricts its ability to invade the epithelial cells for replication [9]. Another major barrier that has been investigated is the midgut escape barrier (MEB) [10,11]. In this case the virus is unable to efficiently disseminate from the midgut following efficient replication in the epithelial cells. The molecular nature of MIB and MEB is still unknown [7,8,12]. Receptor recognition has been suggested to play a role in overcoming MIB for DENV and for Sindbis virus (*Togaviridae*; *Alphavirus*) [7,13,14]. Furthermore, several genomic regions underlying phenotypic variation in midgut infection and midgut escape of DENV2 were revealed by mapping of quantitative trait loci in different *Ae. aegypti* crosses [10,15,16]. Currently, a major research effort aims at identifying the nature of MEB for arboviruses in *Ae. aegypti* by investigating a novel signal transduction pathway that involves a fibroblast growth factor homologue and proteases [17]. Until the advent of highly virulent American-Asian genotype DENV2 in Cuba in 1981, American genotype DENV2 was the predominant genotype of this serotype in the Caribbean, and in Central- and South-America [18]. American genotype DENV2s are generally associated with classical dengue fever (DF) in humans. In contrast, American-Asian genotype DENV2s cause DF but are also associated with a more severe disease outcome, dengue hemorrhagic fever. Interestingly, others have shown that American genotype DENV2 strains are less efficiently transmitted by *Ae. aegypti* than DENV2 strains from Southeast-Asia [19,20]. Less efficient vector transmission of American genotype DENV2 strains has been associated with less efficient dissemination from the mosquito midgut to secondary tissues [19,21]. The DENV2-QR94 strain used in this study was isolated in 1994 from a patient in Quintana Roo, Mexico [22,23]. DENV2-PR159 was isolated in 1969 from a patient in Puerto-Rico and later on cultivated *in vitro* as a vaccine candidate [24]. In this study, we show that DENV2-QR94 and PR159 dissemination from the mosquito midgut is inhibited in the laboratory–adapted Higgs White Eye (HWE) strain of *Ae. aegypti*. Due to a low fidelity replication mechanism depending on the virus-encoded RNA-dependent RNA polymerase (RdRP), DENV populations as well as any other RNA virus populations are generally complex and comprise of a cloud of related genomes, or quasispecies within an infected individual [25]. This quasispecies diversity is important for pathogenesis and adaptation.

Here we isolate midgut escape mutants of DENV2-QR94 and DENV2-PR159 from the viral quasispecies that efficiently overcome the MEB and identify additional amino acid (aa) changes in the genomes of the viral escape mutants that potentially account for their efficient dissemination from the mosquito midgut. Our results demonstrate that the midgut barrier in the mosquito is shaping the quasispecies of DENV2 during the infection process. We show for the first time that the mosquito midgut is a selective sieve for DENV2 in which dissemination-competent viruses are rapidly selected from the viral quasispecies.

## Results

### Phylogenetic characterization of DENV2-QR94 and DENV2-PR159

The PR159 and QR94 strains have been assigned to the American genotype (genotype V) of DENV2 based on 240 bp nucleotide sequences from their E/NS1 gene region [26,27]. Both viruses also had the N390D aa substitution in their E proteins, which is characteristic for American genotype DENV2 [28]. A phylogenetic analysis of DENV2 strains designated as American genotype strains in NCBI GenBank data base showed that the viruses, which were isolated between 1969 and 1998, formed three major clusters. One cluster contained all American genotype strains isolated between 1971 and 1974 in Polynesia/Melanesia (Additional file 1). The second cluster comprised of American genotype strains collected in Puerto-Rico between 1969 and 1977, including PR159. Finally, a third cluster contained American genotype DENV2 strains, which were collected between 1983 and 1998 in Central and South America and included QR94. DENV2-PR159 showed the closest relationship to PR_V3367. Both viruses were isolated in Puerto-Rico in 1969. DENV2-QR94 showed the closest relationship to MX_V3356 isolated in Mexico in 1992.

### American genotype DENV2-QR94 has significantly lower midgut infection and dissemination efficiencies in D2S3 and Chetumal strains of *Ae. aegypti*

**Figure 1:**
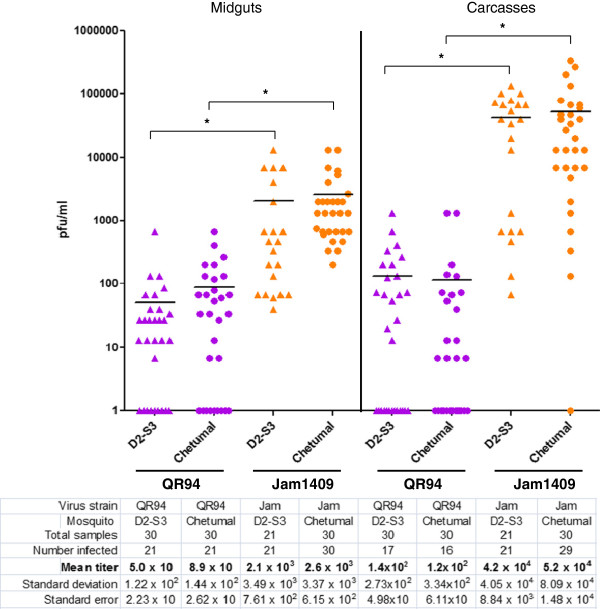
**Oral challenge experiment of*Ae. aegypti*‘D2S3’ and ‘Chetumal’ with DENV2-QR94 and DENV2-Jam1409.** One week post-emergence females of the D2S3 strain (Dengue2 Susceptible on 3 chromosomes strain based on an *Ae. aegypti* ssp. *aegypti* x *Ae. aegypti* ssp. *formosus* hybrid) and Chetumal strain (originating from Yucatan peninsula, Mexico) received artificial bloodmeals containing ~10^7^ pfu/ml DENV2-QR94 (American genotype DENV2 from Quintana Roo, Mexico) or DENV2-Jam1409 (American-Asian genotype DENV2 from Jamaica as control). Viruses had been propagated for 12–14 days in C6/36 cells. Artificial bloodmeals consisted of infected C6/36 cell culture supernatants mixed at a 1:1 ratio with defibrinated sheep blood. Mosquitoes were allowed to feed for 1 h. Virus titers of individual midguts and carcasses were assayed by plaque assays in LLC-MK2 (monkey kidney) cells at 14 days post-bloodmeal (pbm). Bars in the diagram represent mean values. * indicate significant difference between mean virus titers as analyzed on Y = Log(Y) transformed data by ANOVA and Bonferroni’s Multiple Comparison Test.

In an initial experiment, we challenged *Ae. aegypti* mosquitoes of the highly DENV2-competent D2S3 and Chetumal strains with DENV2-QR94. The well-characterized laboratory strain DENV2-Jam1409 was used as a control for comparison. At 14 days pbm, DENV2-QR94 generated midgut infection rates in Chetumal and D2S3 mosquitoes that were significantly lower (70%, p = 0.0019) than those generated by DENV2-Jam1409 (Table 1, Figure 1). Similarly, DENV2-QR94 disseminated from the midguts of both mosquito strains significantly less efficient than the DENV2-Jam1409 control. Mean DENV2-QR94 titers were also significantly lower in midguts and carcasses of both mosquito strains (50–140 pfu/ml for D2S3; 89–120 pfu/ml for Chetumal; p-values: 0.0001-0.0013) than mean titers of the DENV2-Jam1409 control (2.1×10^3^-5.2×10^4^ pfu/ml). No significant difference was observed when comparing mean DENV2-QR94 titers between midguts and carcasses of either mosquito strain. The same observation was made when both mosquito strains were infected with DENV2-Jam1409. Previously, Salazar and colleagues described diminished dissemination rates for DENV2-QR94 in Chetumal mosquitoes when analyzed by immuno-fluorescence assays [21]. Our quantitative assay revealed that not only the midgut dissemination rate of the virus but also its midgut infection rate was significantly reduced in Chetumal and D2S3 mosquitoes.

**Table 1:** **Midgut infection and midgut escape rates of DENV2 strains/isolates QR94, PR159, EM33, EM41, and Jam1409 in*Aedes aegypti*strains D2S3, Chetumal, or HWE at 14 days pbm**

**DENV2 strain/isolate (*Ae. aegypti*strain)**	**No. of mosquitoes**	**Midgut infection rate (%)**	**Midgut dissemination rate (%)**
QR94 (D2S3)	30	70^*a*^	62^*a*^
QR94 (Chetumal)	30	70^*a*^	67^*a*^
Jam1409 (D2S3)	21	100^*b*^	100^*b*^
Jam1409 (Chetumal)	30	100^*b*^	97^*b*^
PR159 (HWE)	30	13^*a*^	3^*a*^
EM41 (HWE)	30	60^*b*^	50^*b*^
Jam1409 (HWE)	30	97^*c*^	77^*b*^
QR94 (HWE)	27	56^*a*^	0^*a*^
EM33 (HWE)	29	34^*a*^	40^*b*^
Jam1409 (HWE)	30	80^*b*^	85^*c*^

Calculation of midgut dissemination rates was based on those individuals whose midguts were DENV2 infected. Letters ^a, b, c^next to numbers represent statistically significant groupings (Fisher's exact test; p-value: <0.05).

### Midgut dissemination of DENV2-QR94 and PR159 is inhibited in the HWE strain of *Ae. aegypti*

**Figure 2:**
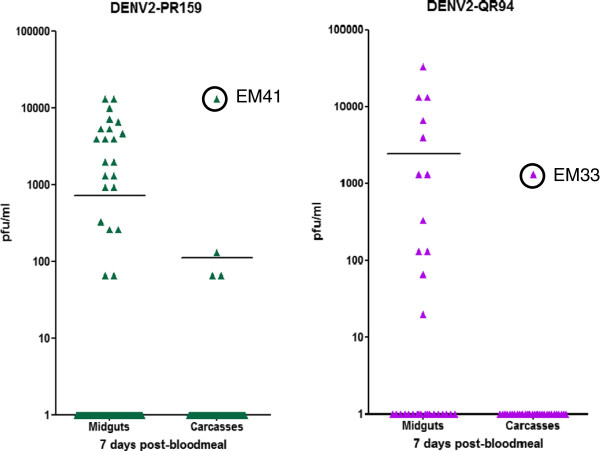
**Isolation of DENV2-PR159 derived viral escape mutant EM41 and of DENV2-QR94 derived EM33.** One week post-emergence, females of the laboratory adapted Higgs White Eye (HWE) strain received artificial bloodmeals containing ~10^7^ pfu/ml DENV2-QR94 (American genotype DENV2 from Quintana Roo, Mexico) or DENV2-PR159 (American genotype DENV2 from Puerto-Rico). Viruses had been propagated for 12–14 days in C6/36 cells. Artificial bloodmeals consisted of infected C6/36 cell culture supernatants mixed at a 1:1 ratio with defibrinated sheep blood. Mosquitoes were allowed to feed for 1 h. Virus titers of individual miguts were assayed by plaque assays in LLC-MK2 (monkey kidney) cells at 7 days post-bloodmeal. Bars in the diagram represent mean values. Numbers of tested individuals to identify EM41 and EM33 were 121 and 31, respectively.

**Figure 3:**
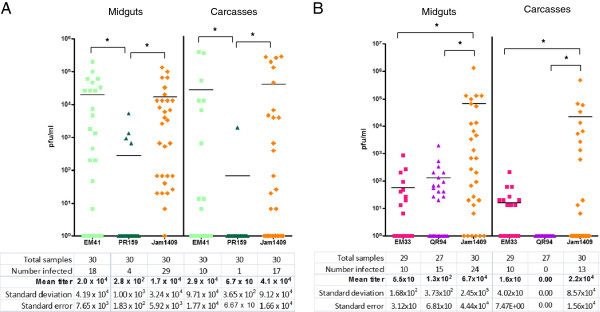
**Oral challenge of*Ae. aegypti*(HWE strain) with DENV2 strains and midgut escape isolates.** One week post-emergence, females of the laboratory adapted Higgs White Eye (HWE) strain received artificial bloodmeals containing **(A)** DENV2-PR159 (American genotype DENV2 from Puerto-Rico), EM41 (midgut escape mutant derived from the PR159 strain), or DENV2-Jam1409 (American-Asian genotype DENV2 from Jamaica as control); **(B)** DENV2-QR94 (American genotype DENV2 from Quintana Roo, Mexico), EM33 (midgut escape mutant derived from the QR94 strain), or DENV2-Jam1409. Viruses had been propagated for 12–14 days in C6/36 cells. Artificial bloodmeals consisted of infected C6/36 cell culture supernatants mixed at a 1:1 ratio with defibrinated sheep blood. Virus titers in the bloodmeals ranged from 7×10^6^-1×10^7^ pfu/ml. Mosquitoes were allowed to feed for 1 h. At 14 days post-bloodmeal (pbm), virus titers of individual midguts and carcasses were assayed by plaque assays in LLC-MK2 (monkey kidney) cells. Bars in the diagram represent mean values. * indicate significant difference between mean titers as analyzed on Y = Log(Y) transformed data by ANOVA and Bonferroni’s Multiple Comparison Test.

After oral acquisition by the laboratory adapted HWE mosquitoes, DENV2-PR159 inefficiently disseminated from the mosquito midgut at 7 or 14 days pbm as determined by plaque assays (Table 1, Figures 2 and 3A). Four out of 121 mosquitoes had disseminated infections at 7 days pbm (Figure 2) and only one disseminated infection (out of 30) was detectable in another independent experiment at 14 days pbm (Figure 3A). Midgut infection rate and mean titer in midgut tissue were significantly lower for DENV2-PR159 (13%, p < 0.0001; 280 pfu/ml; p < 0.0001) when compared to the DENV2-Jam1409 control (Table 1, Figure 3A).

Only a single disseminated infection was detectable in HWE mosquitoes at 7 days pbm and none at 14 days pbm when orally infected with DENV2-QR94 (Table 1, Figures 2 and 3B). Thus, in the HWE strain of *Ae. aegypti* but not in the highly DENV2 competent D2S3 and Chetumal mosquitoes (Figure 1), DENV2-QR94 dissemination was almost completely inhibited. Similar to DENV2-PR159, midgut infection rate and mean midgut virus titer were significantly lower for the QR94 strain (56%, p = 0.0292; 130 pfu/ml; p < 0.0001) in comparison to DENV2-Jam1409 (Table 1, Figures 3A and 3B).

### Isolation of viral midgut escape mutants derived from DENV2-QR94 and PR159

Following oral challenge of HWE mosquitoes with either DENV2-QR94 or DENV2-PR159, we detected high virus titers in two mosquito carcasses at 7 days pbm by plaque assays (Figure 2). We identified one isolate, EM41, from a carcass of a DENV2-PR159 infected mosquito (out of 121 mosquitoes tested), which had a relatively high virus titer (1.3×10^4^ pfu/ml). We also identified an isolate from a carcass of a DENV2-QR94 infected mosquito (out of 31 mosquitoes tested), EM33, which had a virus titer of 1.3×10^3^ pfu/ml. C6/36 cells were infected with virus suspensions of each of the two original carcass samples to amplify the midgut escape mutants by a single passage in cell culture. This ensured that sufficient virus stock was available for the following mosquito challenge and genome sequencing experiments.

### EM41 and EM33 are dissemination-competent variants of their parental DENV2 strains

To three groups of HWE mosquitoes we fed infectious bloodmeals containing DENV2-PR159, EM41, or the American-Asian genotype DENV2-Jam1409 as control and to another three groups we fed bloodmeals containing DENV2-QR94, EM33, or DENV2-Jam1409 (Figures 3A and 3B). In orally challenged HWE mosquitoes, the viral escape mutant EM41 generated mean virus titers in midguts (2.0×10^4^ pfu/ml) and in carcasses (2.9×10^4^ pfu/ml) at 14 days pbm, which were significantly higher (p < 0.0001) than those of the parental DENV2-PR159 (Figure 3A). Midgut infection (60%) and dissemination rates (50%) were also significantly (p < 0.0001) increased for EM41 when compared to DENV2-PR159 (Table 1). However, the midgut infection rate of EM41 was still significantly lower (p = 0.0011) than that of the DENV2-Jam1409 control (97%), whereas the midgut dissemination rates did not differ significantly between the two viruses.

The DENV2-QR94 derived escape variant EM33 generated a midgut dissemination rate of 40% in HWE at 14 days pbm. In contrast, the parental DENV2-QR94 was not able to disseminate from the midgut (Table 1, Figure 3B). The EM33 midgut infection (34%, p = 0.0006) and dissemination rates (40%, p = 0.004) were still significantly lower than those observed for DENV2-Jam1409. Likewise, mean EM33 titers in midguts (55 pfu/ml, p < 0.0001) and carcasses (16 pfu/ml, p = 0.0006) were significantly lower in comparison to the mean titers generated by the Jam1409 control. Taken together, our data show that EM41 and EM33 are dissemination-competent variants of their parental virus strains, DENV2-PR159 and DENV2-QR94, respectively.

### DENV2-QR94 and DENV2-PR159 have different replication efficiencies in mosquito tissue following intrathoracic injection

**Figure 4:**
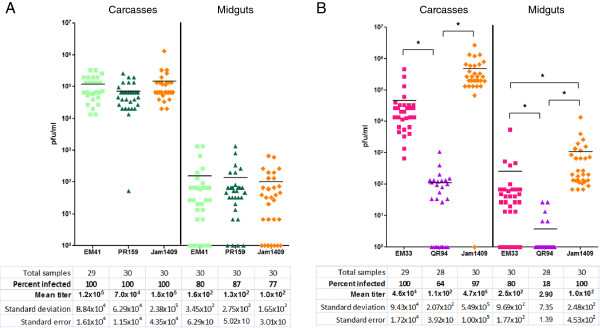
**Intrathoracic injection of DENV2 strains and midgut escape isolates into HWE mosquitoes.(A)** Intrathoracic injection of DENV2-PR159 (American genotype DENV2 from Puerto-Rico), EM41 (midgut escape mutant derived from the PR159 strain), and DENV2-Jam1409 (American-Asian genotype DENV2 from Jamaica as control) into females of the laboratory adapted Higgs White Eye (HWE) strain. Viruses had been propagated for 12–14 days in C6/36 cells. Prior to injection, virus-cell suspensions had titers ranging from 5x10^5^ pfu/ml to 7x10^6^ pfu/ml. **(B)** Intrathoracic injection of DENV2-QR94, (American genotype DENV2 from Quintana Roo, Mexico), EM33 (midgut escape mutant derived from the QR94 strain), and DENV2-Jam1409 into HWE females. Virus-cell suspensions had titers of ~10^6^ pfu/ml. Virus titers of individual midguts and carcasses were assayed by plaque assays in LLC-MK2 (monkey kidney) cells at 7 days post-bloodmeal (pbm). Bars in the diagram represent mean values. * indicate significant difference between mean virus titers as analyzed on Y = Log(Y) transformed data by ANOVA and Bonferroni’s Multiple Comparison Test.

We intrathoracically injected DENV2-QR94, PR159 and their midgut escape mutants EM33 and EM41, respectively into the HWE strain of *Ae. aegypti* to assess whether the viruses had similar replication efficiencies in mosquito tissue outside the midgut. Following intrathoracic injection of DENV2-PR159, its replication efficiency in mosquito carcasses (and midguts) was similar to that of EM41 and DENV2-Jam1409 (Figure 4A). In contrast, mosquitoes intrathoracically injected with DENV2-QR94 produced a significantly lower mean virus titer (1.1x10^2^ pfu/ml; p < 0.0001) in carcasses of HWE than mosquitoes injected with EM33 or the DENV2-Jam1409 control (Figure 4B). Following injection into the mosquito thorax, the QR94 strain invaded midgut tissue with a significantly lower efficiency (18%; p < 0.0001) than either EM33 or DENV2-Jam1409. These data indicate that the inability of DENV2-PR159 to productively infect the mosquito was caused by the virus’ inability to overcome the MEB rather than poor replication efficiency. In contrast, DENV2-QR94 not only was confronted with a strong MEB in *Ae. aegypti* but also had a significantly lower replication efficiency in secondary tissue outside the midgut.

### Unique amino acid substitutions in the genomes of the dissemination incompetent DENV2 strains QR94 and PR159

DENV2-QR94 and its closest relative, Mexican strain MX_V3356 (Additional file 1), differed from each other in 24 aa residues. DENV2-PR159 and its close relative PR_V3367 differed from each other in five aa residues. DENV2-QR94 and DENV2-PR159 diverged at 26 aa positions. The QR94 strain had seven aa residues and the PR159 strain had four aa residues that were not found in 100 of the most closely related DENV2 isolates listed in NCBI GenBank based on BLAST searches (Table 2). In DENV2-QR94, two of the seven unique aa changes were present in structural proteins, E89G in prM and T55S in domain II of E. Other unique aa changes occurred in NS1, NS2a, the helicase domain of NS3 (ranging from aa position 179–618), the methyltransferase domain of NS5 (aa position 1–272) and the finger domain of the RdRP of NS5 (aa position 273–511). DENV2-PR159 had a unique aa change in domain II of E, which was E202K. The other three unique aa changes occurred in NS1, NS2a, and the thumb domain (aa position 627–900) of the RdRP of NS5.

**Table 2:** Unique amino acid changes in American genotype DENV2 (parental strains QR94 and PR159, PR159-derived midgut escape isolate EM41 and QR94-derived midgut escape isolate EM33) based on BLAST searches in NCBI GenBank

**Virus**	**C**	**prM**	**E**	**NS1**	**NS2a**	**NS2b**	**NS3**	**NS4a**	**NS4b**	**NS5**
QR94		E89G	T55S	I247Y	T25S		A373T			N174D
				L247Y						K469R
				F247Y						
EM33		E89G	T55S	I247Y	T25S		A373T			N174D
			**E202K**	L247Y						K469R
				F247Y						
PR159			E202K	M123T	A88T					S631N
EM41			**Q77E**	M123T	A88T		**E93D**			S631N
			E202K							

In bold: additional amino acid substitutions identified in the midgut escape isolates EM33 and EM41, which were absent in the parental viruses.

### Amino acid substitutions in the genomes of EM33 and EM41 associated with efficient midgut dissemination phenotypes

All aa substitutions in the structural proteins of the escape mutants EM33 and EM41 resulted in a change of charge (Table 2). Escape mutant EM33 had a single additional aa substitution from negative to positive charge, E202K. Residue 202 is located within domain II of the E protein, which allows protein dimerization and contains the fusion peptide. DENV2-PR159 had the identical E202K aa change in its E protein even though the virus was unable to disseminate from the mosquito midgut. The PR159-derived escape mutant EM41 had two compensatory aa substitutions, E93D in NS3 and Q77E in domain II of the E protein in close proximity (21 aa) to the fusion peptide. Q77E resulted in an aa change from positive to negative charge. The E93D aa substitution was located in the protease domain of NS3 and also resulted in a change of charge from negative to positive polarity.

## Discussion

Apart from causing reduced virulence levels in the vertebrate host, American genotype DENV2 strains have been reported to be less efficiently transmitted by *Ae. aegypti*[19,20,29,30]. Specifically, Armstrong and Rico-Hesse (2003) reported that six American genotype DENV2 strains isolated from Trinidad, Venezuela, Mexico, and Peru poorly disseminated from midguts of two different *Ae. aegypti* strains when compared to dissemination rates of nine DENV2 strains from Southeast-Asia [19]. None of the six American genotype DENV2 strains used in their study contained the unique aa substitutions described in DENV2-QR94 and DENV2-PR159 [29]. Others have speculated that American genotypes have lower efficiency in formation of replicative intermediates than viruses from Southeast-Asia, which leads to lower fitness levels of American genotype viruses in mosquitoes and vertebrate hosts [30]. This could be the case for DENV2-QR94, which showed reduced replication efficiencies in midgut tissue following oral acquisition and in secondary tissue following intrathoracic injection. Intrathoracically injected DENV2-PR159, however, generated a mean virus titer in HWE mosquitoes that was comparable to that of American-Asian genotype DENV2-Jam1409. Immuno-fluorescence studies suggested earlier that DENV2-QR94 poorly disseminated from midguts of Chetumal mosquitoes at 4–7 days pbm by generating significantly fewer head tissue infections than American-Asian genotype DENV2 strains [21]. Here, we show diminished dissemination of DENV2-QR94 from midguts of Chetumal mosquitoes as well as from midguts of two other DENV2-competent mosquito strains, D2S3 and HWE. Similar observations were made when assessing the vector competence of *Ae. aegypti* (strain HWE) for American genotype DENV2-PR159. Our study demonstrates that the mosquito midgut is a selective sieve for DENV2 from which we could readily select dissemination-competent virus variants. We isolated from mosquitoes two viral midgut escape mutants, EM33 derived from DENV2-QR94 and EM41 of DENV2-PR159 that efficiently disseminated from the midgut.

The low-passage DENV2-QR94 virus had seven aa changes that have not been found in other wild-type DENV2 previously sequenced. Two of those aa changes occurred in structural proteins, E89G in prM and T55S in E. prM acts as a chaperone for E during virion assembly and maturation by preventing E from undergoing premature conformational changes during excretion through the trans-Golgi network at low pH [31]. The E89G aa mutation in prM leads to a change of charge at position X of the four residue furin cleavage site R-X-K/R-R [32]. Furin cleavage occurs late during virion maturation and is involved in E rearrangement and overall virus infectivity. Thus, the E89G mutation may impact efficient furin cleavage of prM. Interestingly, Kelly and colleagues observed an E89G mutation in prM after 30 serial passages of the attenuated DENV2 S16803 Thailand vaccine strain in primary dog kidney cells [32]. The midgut escape mutant EM33 had one additional aa substitution, E202K in the viral E protein, resulting in a change of charge from negative to positive polarity. The 202 residue is located within the fg loop, which is part of the hinge region connecting E domain I with domain II [33]. An aa substitution at this position is assumed to affect virus pathogenicity [32]. The E202K mutation in E has also been reported in DENV2-S16803 after 10 serial passages in primary dog kidney cells [32]. Importantly, the high passage DENV2-PR159 strain had the identical E202K mutation seen in EM33, although the lysine in this position in DENV2-PR159 E protein did not lead to virus dissemination from the midgut as observed for EM33.

Two aa changes in EM41 were associated with dissemination from the midgut, Q77E and E93D. Q77E leads to a charge change in a residue of domain II of the E protein, 21 residues upstream of the fusion peptide. The negative charge change of Q77E may have compensated for the positive charge change of E202K in the PR159 strain. The other aa substitution, E93D, occurred in the proteinase encoding domain of NS3. Both Q77E and E93D have not been described before in DENV2. Based on the observations made by Kelly and colleagues, it may be possible that aa substitutions E89G in DENV2-QR94 and E202K in the PR159 strain are a result of virus passage in cell culture and might not have been present in the genome sequences of the original virus samples that had been collected from infected patients [32].

Recently, we have shown that transgene-mediated suppression of the antiviral RNA interference pathway (RNAi) in *Ae. aegypti* significantly increased mean DENV2-QR94 titers in midguts but did not lead to increased dissemination efficiency [34]. Thus, the inability of DENV2-QR94 to disseminate from the mosquito midgut did not depend on the RNAi response of the mosquito. However, at this point it cannot be excluded that other innate immune pathways of the mosquito may contribute to the defect in dissemination of QR94 [35,36]. Furthermore, we speculate that the single aa substitutions in prM (E89G) and E (E202K) negatively affect the ability of DENV2 virions to attach to recognition sites at the midgut basal lamina that would enable the virions to traverse this barrier. Alternatively, the mutations could hamper efficient virion maturation and/or conformational changes of virion surface structures that prevent the virus from escaping from the midgut. DENV2-QR94 not only failed to efficiently disseminate from the mosquito midgut but also produced significantly lower mean virus titers in midguts and secondary tissue in comparison to DENV2-PR159 or the DENV2-Jam1409 control, implying that the overall replication potential of the virus was diminished. Since DENV2-QR94 also had two unique aa substitutions in the polymerase encoding NS5, it is possible that these residues accounted for the reduced replication efficiency of the virus. However, it will be important to confirm our observations concerning aa substitutions affecting midgut escape of DENV2 by mutational analysis using an American genotype DENV2-derived infectious cDNA clone.

Previously, it was suggested that point mutations in the 3′UTR of American genotype DENV2 strain QR94 might contribute to its reduced midgut dissemination and replication efficiency [21]. The 3′UTRs (453 nt) of DENV2-QR94 and DENV2-PR159 vary in five nucleotide positions (Additional file 2). When compared to the 3′UTR of American-Asian genotype DENV2-Jam1409, the variation amounts to 26 positions including a nine nucleotide insertion. However, DENV2-PR159 and EM41 have identical 3′UTR nucleotide sequences and the 3′UTR of DENV2-QR94 and EM33 differ in a single nucleotide. Thus, mutations in the 3′UTR may not be seen as a likely cause for the poor midgut dissemination efficiency of the parental viruses.

## Conclusions

We have shown that two American genotype DENV2 strains, QR94 and PR159 are inhibited in their ability to disseminate from the midgut of *Ae. aegypti,* which led to the selection of two midgut escape mutants of the viruses, EM33 and EM41 from the viral quasispecies. This study identified dissemination-deficient DENV2 strains and dissemination-competent escape mutants. Both viral escape mutants had (charge-changing) aa changes in structural proteins and/or in NS3. The generation of the escape mutants occurred after a single passage in the mosquito, indicating that the midgut of *Ae. aegypti* acts as a selective sieve for DENV2 in which genetically distinct, dissemination-competent virus variants are rapidly selected from the viral quasispecies to be transmitted to vertebrates. The escape mutants EM33 and EM41 and the parental strains DENV2-QR94 and DENV2-PR159, respectively, are potentially important viruses for defining arbovirus midgut escape mechanisms in *Ae. aegypti*.

## Methods

### Mosquitoes

*Ae. aegypti* strains of this study were the Chetumal strain of the Yucatan peninsula, Mexico [37], the D2S3 (Dengue2 Susceptible on 3 chromosomes) strain, which is based on an *Ae. aegypti* ssp. *aegypti* (San Juan, Puerto Rico) x *Ae. aegypti* ssp. *formosus* (Ibo, Nigeria) hybrid [15], and the RexD (Puerto Rico)-derived Higgs White Eye (HWE) strain, which is frequently used for germline transformation experiments [38,39]. The Chetumal and D2S3 mosquito strains are highly DENV2 competent. Mosquitoes of all three strains were reared under identical conditions (28°C temperature, 78% humidity, 12 h light/12 h dark cycle) in a BSL2 insectary. For colony maintenance, mosquitoes received artificial bloodmeals consisting of citrated sheep blood.

### Viruses

DENV2-PR159 was obtained from the Centers for Disease Control and Prevention (USA). The virus, originally isolated in 1969, has an unknown (high) passage history in C6/36 *Ae. albopictus* cells. Virus stocks for mosquito challenge experiments had titers of 6.8×10^6^ pfu/ml. The QR94 strain of DENV2 was isolated in 1994 from a patient in Quintana Roo, Mexico [22,23]. Since its isolation, the virus has been passed 4× in C6/36 cells [21]. The original virus stock had a titer of 7×10^5^ pfu/ml. The American-Asian genotype DENV2-Jam1409 is derived from a full-length infectious cDNA clone [40]. Stocks of the virus were generated in C6/36 cells and had titers ranging from of 1×10^6^-2×10^7^ pfu/ml. Virus stocks of midgut escape mutants were generated by a single passage of sterile-filtered mosquito carcass suspensions in C6/36 cells at a multiplicity of infection (m.o.i.) of 0.01. Twelve to 14 days post-infection, cells and medium were collected and used as virus stocks for mosquito challenge experiments and cDNA sequencing.

### Phylogenetic analysis of American genotype DENV2 strains

DENV2 strains included in the phylogenetic analysis have the following NCBI GenBank accession numbers: D2/PF/UH50/1972 (HM582108.1), D2/PF/UH57/1971 (HM582109), D2/FJ/UH21/1971 (HM582099), D2/FJ/UH22/1971 (HM582101.1), D2/FJ/UH40/1971 (HM582100.1), D2/NC/UH37/1971 (HM582102.1), D2/NC/UH97/1972 (HM582103.1), D2/TO/UH00/1973 (HM582110), D2/TO/UH04/1974 (HM582117), D2/TO/UH16/1974 (HM582111), D2/TO/UH19/1974 (HM582112), D2/TO/UH39/1974 (HM582114), D2/TO/UH44/1974 (HM582115), D2/TO/UH94/1974 (HM582116.1), D2/AS/UH77/1972 (HM582104), D2/AS/UH79/1972 (HM582105), D2/AS/UH85/1972 (HM582106), DENV-2/PR/BID-V3367/1969 (GQ868600), Dengue virus type 2 isolate 1328 (EU056812), DENV-2/MX/BID-V3354/1983 (GQ868588), DENV-2/VE/BID-V3366/1987 (GQ868599), DENV-2/HN/BID-V2945/1984 (FJ898449), DENV-2/MX/BID-V3356/1992 (GQ868590), Dengue virus type 2 isolate IQT-1950 (EU056811), Dengue virus type 2 strain IQT2913 (AF100468.1), Dengue virus type 2 strain IQT1797 (AF100467), Dengue virus type 2 Jamaica/N.1409 (M20558). Full-length nucleotide sequences of DENV2-QR94 and DENV2-PR159 are available at NCBI GenBank under accession numbers JX966379 and JX966380, respectively. Nucleotide sequences were automatically aligned with ClustalW in MEGA5 [41] and manually inspected in MEGA5. The phylogeny was inferred under maximum likelihood in MEGA5 using the Tamura-Nei model [42]. Bootstrapping was conducted in MEGA5 with 1000 replicates.

### Oral virus challenge experiments and intrathoracic virus injections into mosquitoes

Virus challenge experiments with mosquitoes were performed as described before [38,43]. Briefly, DENV2 stocks (Jam1409, PR159, QR94, EM33, EM41) were cultivated in *Ae. albopictus* (C6/36) cells by infecting cells at 0.01 multiplicity of infection (m.o.i.), changing the culture medium at 6 days post-infection and maintaining the cells until 12–14 days post-infection. One-week-old females of the HWE, D2S3, or Chetumal strains received artificial, DENV2 containing bloodmeals, which consisted of 50% C6/36 cell suspension, 50% defibrinated sheep blood and 1 mM ATP. Glass feeders were covered with hog gut and connected to a water jack that had a temperature of 37°C. One feeder was placed on each mosquito carton containing 100 females, which had been starved for around 18 h prior to bloodfeeding. Each glass feeder was then filled with 2 ml cell culture-blood mixture. Mosquitoes were allowed to feed for about 1 h. Only blood-engorged mosquitoes were used in subsequent experiments. Virus titers in the bloodmeals ranged from 7×10^6^-1×10^7^ pfu/ml. Intrathoracic injections of DENV2s were conducted to assess virus replication in mosquito tissues outside the midgut. Seven-day-old females were injected each with 150–200 nl of the virus-cell suspensions with titers of ~1×10^6^ pfu/ml (QR94, EM33, Jam1409), 5×10^5^ pfu/ml (EM41), and 7×10^6^ pfu/ml (PR159).

### Virus detection

DENV2 titers of single female midguts and carcasses were analyzed by plaque assays at 7 and 14 days pbm using LLC-MK2 monkey kidney cells. DENV2-infected samples were homogenized in 0.5 ml 7% FBS-complemented DMEM medium before being sterile-filtered with Acrodisc HT Tuffryn 0.2 μm syringe filters (Pall Life Sciences, East Hills, NY). LLC-MK2 cells were seeded into 24-well plates and left for three days to achieve confluence. Cells were infected with 10-fold serial dilutions of individual sample homogenates and then incubated for 1 h at 37°C before being overlaid with an agarose nutrient mixture [1x Medium 199 (Sigma-Aldrich, St. Louis, MO), 10% FBS, 4% NaHCO_3_, 0.5% MEM vitamins, 0.5% MEM amino acids (Mediatech Inc., Manassas, VA); 1% Hanks-DEME medium]. Following incubation at 37°C for 12 days, cells were then stained with MTT (3-[4,5-dimethylthiazol-2-yl]-2,5-diphenyltetrazolium bromide) (Sigma-Aldrich, St. Louis, MO). After incubation at 37°C for 24 h the number of plaques was counted for each sample. Virus titers of individual mosquitoes were calculated as plaque forming units (pfu)/ml.

### RT-PCR and cDNA sequencing

Total RNA was extracted from 1–2 ml aliquots of DENV2-infected C6/36 cell suspensions using TRIzol LS reagent (Invitrogen, CA). RT-PCR was conducted using the Superscript II One-Step RT-PCR with Platinum Taq kit (Invitrogen, CA) according to the manufacturer’s instructions. Full-length cDNAs of the different DENV2 strains were sequenced. Therefore, 23 sets of PCR primers were designed to generate PCR products of around 500 bp in size that each overlapped by 50–100 bp. Viral cDNA encoding the 5′ and 3′ UTRs were amplified using 5′ and 3′RACE Systems for Rapid Amplification of cDNA Ends (Invitrogen, CA) according to the manufacturer’s instructions. PCR products were purified using the Qiagen PCR Purification kit (Qiagen, Valencia, CA) and then directly sequenced using an ABI-3130×L Genetic Analyzer. Presence of mutations was confirmed by repeated sequencing using different cDNA sources.

### Statistical analysis

Statistical analysis was performed using the GraphPad Prism software package (version 5.04). Raw plaque data were Y = Log(Y) transformed to ensure homogenous variances among data points and then analyzed by the ANOVA procedure followed by Bonferroni’s Multiple Comparison Test. To compare virus titers only positive values were included in the statistical analysis. Midgut infection and dissemination rates were analyzed using Fisher’s Exact Test.

## Competing interests

The authors declare that they have no competing interests.

## Authors’ contributions

CCHK conceived and designed the experiments, performed virus challenge experiments of mosquitoes, conducted virus detection assays, and analyzed the data. JBD assisted with virus challenge experiments, performed sample preparations, and assisted with virus detection assays. NLH conducted the phylogenetic analysis. KEO discussed the experimental design, analyzed the data, and helped to prepare the manuscript. AWEF is PI of the NIH grants that made these studies possible, conceived and designed the experiments, analyzed the data, and prepared the manuscript. The authors approve submission of the manuscript to Virology Journal. All authors read and approved the final manuscript.

## Supplementary Material

Additional file 1 **Maximum likelihood phylogeny for American genotype DENV2 strains including DENV2-QR94 and DENV2-PR159.** DENV2 viruses originated from Central-America, South-America, the Caribbean, and Polynesia/Melanesia. DENV2-Jam1409, belonging to the American-Asian genotype, is included for comparison. Bootstrap values greater than 70 are shown and indicate support as a percent of 1000 replicates. Scale bar represents 0.01 substitutions per site. Click here for file

Additional file 2 **Nucleotide alignments of the 453 nt 3′UTRs of American genotype DENV2-QR94, PR159, EM33, and EM41.** Mismatches are highlighted in red. Click here for file
