# Supplementary material for: Isolation of midgut escape mutants of two American genotype dengue 2 viruses from Aedes aegypti
Source: Virol J. 2013 Aug 12;10:257. doi: 10.1186/1743-422X-10-257 (PMC3751248; doi:10.1186/1743-422X-10-257)
Supplement: Additional file 1 — Maximum likelihood phylogeny for American genotype DENV2 strains including DENV2-QR94 and DENV2-PR159. DENV2 viruses originated from Central-America, South-America, the Caribbean, and Polynesia/Melanesia. DENV2-Jam1409, belonging to the American-Asian genotype, is included for comparison. Bootstrap values greater than 70 are shown and indicate support as a percent of 1000 replicates. Scale bar represents 0.01 substitutions per site. [file 1743-422X-10-257-S1.pdf]

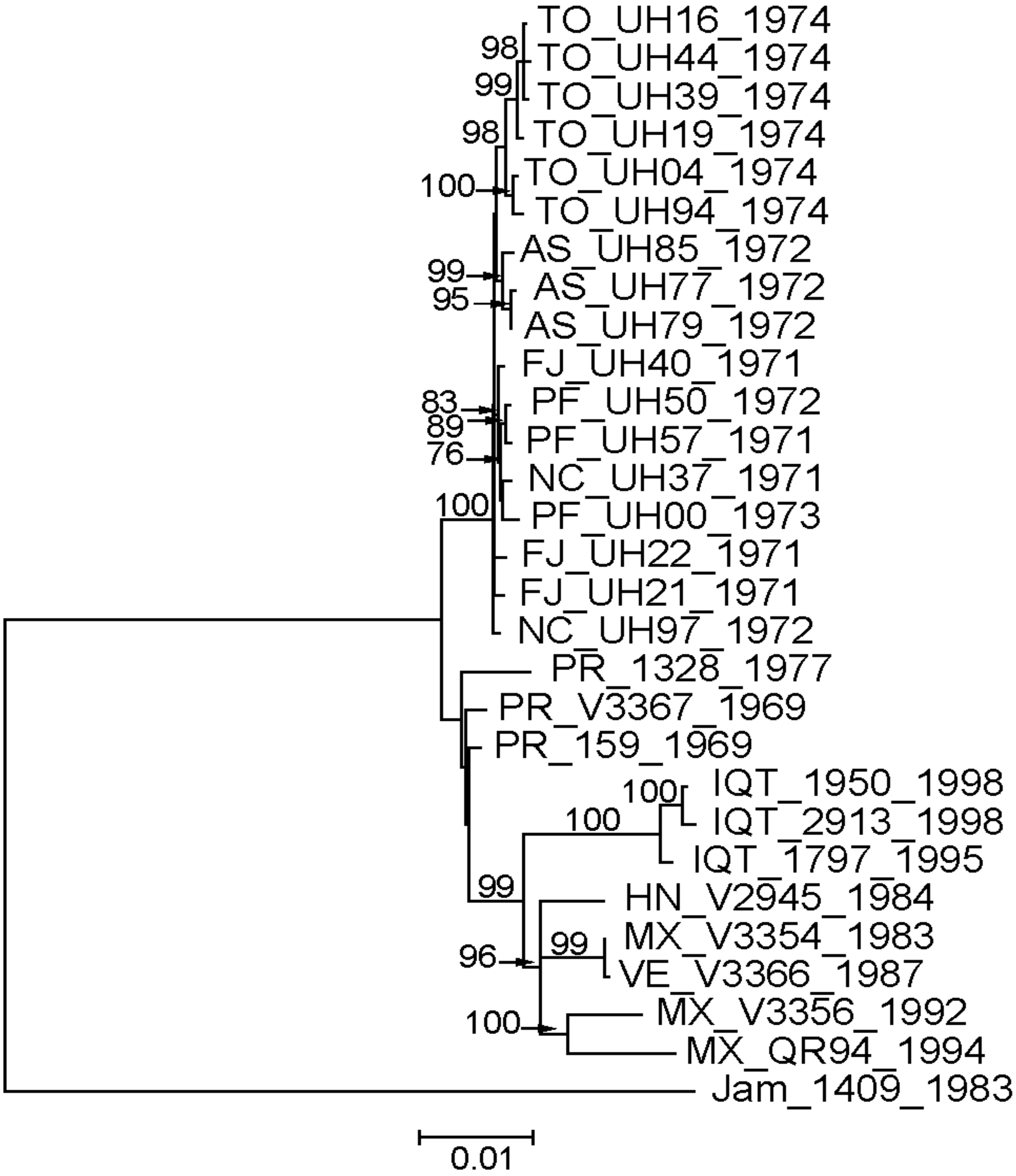

Additional file 1. Maximum likelihood phylogeny for American genotype DENV2 strains including DENV2-QR94 and DENV2-PR159. DENV2 viruses originated from Central-America, South-America, the Caribbean, and Polynesia/Melanesia. DENV2-Jam1409, belonging to the American-Asian genotype, is included for comparison. Bootstrap values greater than 70 are shown and indicate support as a percent of 1000 replicates. Scale bar represents 0.01 substitutions per site.
