# Supplementary material for: Isolation of midgut escape mutants of two American genotype dengue 2 viruses from Aedes aegypti
Source: Virol J. 2013 Aug 12;10:257. doi: 10.1186/1743-422X-10-257 (PMC3751248; doi:10.1186/1743-422X-10-257)
Supplement: Additional file 2 — Nucleotide alignments of the 453 nt 3′UTRs of American genotype DENV2-QR94, PR159, EM33, and EM41. Mismatches are highlighted in red. [file 1743-422X-10-257-S2.pdf]

```
QR94   GTTTTGTGGTAGAAAAACATGAGACAGAACAAAAGTCAGGTCGGATTAAGCCATAGTACGGGAAAAACTATGCTA
EM33   GTTTTGTGGTAGAAAAACATGAGACAGAACAAAAGTCAGGTCGGATTAAGCCATAGTACGGGAAAAACTATGCTA

QR94   CCTGTGAGCCCCGTCCAAGGACGTTAAAAGAAGTCAGGCCACTTTGATGCCATAGCTTGAGCAAACAGTGCAGCC
EM33   CCTGTGAGCCCCGTCCAAGGACGTTAAAAGAAGTCAGGCCACTTTGATGCCATAGCTTGAGCAAACAGTGCAGCC

QR94   TGTAGCTCCACCTGAGAAGGTGTAAAAAATCCGGGAGGCCACAGACCATGGAAGCTGTACGCATGGCGTAGTGGA
EM33   TGTAGCTCCACCTGAGAAGGTGTAAAAAATCCGGGAGGCCACAAACCATGGAAGCTGTACGCATGGCGTAGTGGA

QR94   CTAGCGGTTAGAGGAGACCCCTCCCTTTCAGATCGCAGCAACAATGGGGGCCCAAGGTGAGATGAAGCTGTAGTC
EM33   CTAGCGGTTAGAGGAGACCCcTCCCTTTCAGATCGCAGCAACAATGGGGGCCCAAGGTGAGATGAAGCTGTAGtC

QR94   TCACTGGAAGGACTAGAGGTTAGAGGAGACCCCCCCAAGACAAAAAACAGCATATTGACGCTGGGAAAGACCAGA
EM33   TCACTGGAAGGACTAGAGGTTAGAGGAGACCCCCCCAAAACAAAAAACAGCATATTGACGCTGGGAAAGACCAGA

QR94   GATCCTGCTGTCTCCTCAGCATCATTCCAGGCACAGGACGCCAGAAAATGGAATGGTGCTGTTGAATCAACAGGT
EM33   GATCCTGCTGTCTCCTCAGCATCATTCCAGGCACAGGACGCCAGAAAATGGAATGGTGCTGTTGAATCAACAGGT

QR94   TCT
EM33   TCT


PR159  GTTTTGTGGTAGAAAAACATGAGACAGAACAAAAGTCAGGTCGGATTAAGCCATAGTACGGGAAAAACTATGCTA
EM41   GTTTTGTGGTAGAAAAACATGAGACAGAACAAAAGTCAGGTCGGATTAAGCCATAGTACGGGAAAAACTATGCTA

PR159  CCTGTGAGCCCCGTCCAAGGACGTTAAAAGAAGTCAGGCCACTTTGATGCCATAGCTTGAGCAAACAGTGCAGCC
EM41   CCTGTGAGCCCCGTCCAAGGACGTTAAAAGAAGTCAGGCCACTTTGATGCCATAGCTTGAGCAAACAGTGCAGCC

PR159  TGTAGCTCCACCTGAGAAGGTGTAAAAAATCCGGGAGGCCACAGACCATGGAAGCTGTACGCATGGCGTAGTGGA
EM41   TGTAGCTCCACCTGAGAAGGTGTAAAAAATCCGGGAGGCCACAAACCATGGAAGCTGTACGCATGGCGTAGTGGA

PR159  CTAGCGGTTAGAGGAGACCCCTCCCTTTCAGATCGCAGCAACAATGGGGGCCCAAGGTGAGATGAAGCTGTAGTC
EM41   CTAGCGGTTAGAGGAGACCCcTCCCTTTCAGATCGCAGCAACAATGGGGGCCCAAGGTGAGATGAAGCTGTAGtC

PR159  TCACTGGAAGGACTAGAGGTTAGAGGAGACCCCCCCAAAACAAAAAACAGCATATTGACGCTGGGAAAGACCAGA
EM41   TCACTGGAAGGACTAGAGGTTAGAGGAGACCCCCCCAAAACAAAAAACAGCATATTGACGCTGGGAAAGACCAGA

PR159  GATCCTGCTGTCTCCTCAGCATCATTCCAGGCACAGGACGCCAGAAAATGGAATGGTGCTGTTGAATCAACAGGT
EM41   GATCCTGCTGTCTCCTCAGCATCATTCCAGGCACAGGACGCCAGAAAATGGAATGGTGCTGTTGAATCAACAGGT

PR159  TCT
EM41   TCT


QR94   GTTTTGTGGTAGAAAAACATGAGACAGAACAAAAGTCAGGTCGGATTAAGCCATAGTACGGGAAAAACTATGCTA
PR159  GTTTTGTGGTAGAAAAACATGAAACAAAACAGAAGTCAGGTCGGATTAAGCCATAGTACGGGAAAAACTATGCTA

QR94   CCTGTGAGCCCCGTCCAAGGACGTTAAAAGAAGTCAGGCCACTTTGATGCCATAGCTTGAGCAAACAGTGCAGCC
PR159  CCTGTGAGCCCCGTCCAAGGACGTTAAAAGAAGTCAGGCCACTTTGATGCCATAGCTTGAGCAA-CTGTGCAGCC

QR94   TGTAGCTCCACCTGAGAAGGTGTAAAAAATCCGGGAGGCCACAGACCATGGAAGCTGTACGCATGGCGTAGTGGA
PR159  TGTAGCTCCACCTGAGAAGGTGTAAAAAATCCGGGAGGCCACAAACCATGGAAGCTGTACGCATGGCGTAGTGGA
```

```
QR94    CTAGCGGTTAGAGGAGACCCCTCCCTTTCAGATCGCAGCAACAATGGGGGCCCAAGGTGAGATGAAGCTGTAGTC
PR159   CTAGCGGTTAGAGGAGACCCCTCCCTTACAGATCGCAGCAACAATGGGGGCCCAAGGTGAGATGAAGCTGTAGTC

QR94    TCACTGGAAGGACTAGAGGTTAGAGGAGACCCCCCCAAGACAAAAAACAGCATATTGACGCTGGGAAAGACCAGA
PR159   TCACTGGAAGGACTAGAGGTTAGAGGAGACCCCCCCAAAACAAAAAACAGCATaTTGACGCTGGGAAAGACCAGA

QR94    GATCCTGCTGTCTCCTCAGCATCATTCCAGGCACAGGACGCCAGAAAATGGAATGGTGCTGTTGAATCAACAGGT
PR159   GATCCTGCTGTCTCCTCAGCATCATTCCAGGCACAGGACGCCAGAAAATGGAATGGTGCTGTTGAATCAACAGGT

QR94    TCT
PR159   TCT
```

**Additional file 2:** Nucleotide alignments of the 453 nt 3'UTRs of American genotype DENV2-QR94, PR159, EM33, and EM41. Mismatches are highlighted in red.
